# ID 92 - Análises Econômicas da Donepezila Isolada ou em Combinação à Memantina para Tratamento de Pacientes com Doença de Alzheimer Grave no SUS

**DOI:** 10.5327/2237-9622.2025.v34s1.92

**Published:** 2025-11-25

**Authors:** Francino Machado de Azevedo, Layssa Andrade Oliveira, Haliton Alves Oliveira, Rosa Camila Lucchetta

**Keywords:** donepezila, análise de custo-efetividade, doença de Alzheimer, avaliação em saúde, SUS

## Abstract

**Introdução:**

A donepezila é um inibidor de aceticolinesterase com indicação para tratar efeitos comportamentais e cognitivos da doença de Alzheimer (DA), sendo o único representante da classe com indicação para todos os estágios da doença. Todavia, para pacientes com DA que progridem ou são diagnosticados com a doença já em estágio grave não dispõe de recomendação para uso no SUS. Considerando as recomendações clínicas que sustentam sua ampliação (maior eficácia quando combinada à memantina e ausência de diferença estatística quando em uso isolado), este estudo estimou os resultados econômicos de uma possível incorporação da donepezila no SUS.

**Método:**

Avaliação econômica foi realizada considerando duas abordagens (custo-efetividade, ACE e custo-minimização, ACM), a depender da comparação. Para a ACE adotou-se um modelo de Markov com horizonte temporal de 14 ciclos anuais (baseada na expectativa de vida do paciente diagnosticado com DA no Brasil). Os desfechos incluíram anos de vida ajustados pela qualidade (QALY) e anos de vida (AV) para ACE e diferença de custo para ACM. Em ambas as análises, foram incluídos custos médicos diretos no modelo e utilizado fonte oficial do SUS para apuração. Análises de sensibilidade probabilística e determinística univariada foram performadas.

**Resultados:**

O tratamento combinado de donepezila + memantina (combinação livre) foi associado a um maior benefício (QALY ganho de 0,74) e custo total de tratamento (R$ 9.307) quando comparado à memantina isolada, apresentando razão de custo-efetividade incremental (RCEI) de R$ 6.187/QALY ganho. As análises de sensibilidade probabilísticas indicam que em 100% das simulações os tratamentos contendo donepezila podem ser custo-efetivos para o SUS e as análises determinísticas mostraram que os parâmetros mais influentes foram o preço da combinação livre de donepezila e memantina, mortalidade nos grupos tratados com memantina e donepezila + memantina, custo do tratamento com memantina isolada. Entretanto, em nenhum dos extremos de variação as RCEI ultrapassaram o limiar de disposição a pagar do SUS. A análise de custo-minimização mostrou que a preferência da donepezila à memantina pode levar a redução de custo de R$ 65,70 por paciente atendido no SUS.

**Conclusão:**

Em um cenário de optar pela donepezila ou memantina, a escolha da primeira pode levar a redução do custo de tratamento do paciente com DA grave para o SUS. A ACE revela que ao considerar o limiar de custo-efetividade adotado no SUS, a associação das tecnologias é custo-efetiva.

